# Supplementary material for: Ultrastructural Abnormalities in Induced Pluripotent Stem Cell-Derived Neural Stem Cells and Neurons of Two Cohen Syndrome Patients
Source: Cells. 2023 Nov 25;12(23):2702. doi: 10.3390/cells12232702 (PMC10705360; doi:10.3390/cells12232702)
Supplement: Supplementary file 1 [file cells-12-02702-s001.zip › cells-2674950-supplementary.pdf]

Supplementary Information

Table S1. Antibodies used for immunocytochemistry.

Antibodies	Host	Dilution	Producer, cat#
Golgin 97	Rabbit	1:50	Thermo Fisher Scientific, PA5-30048
Tubb3	Mouse	1:500	Covance, #MMS-435P
Pax6	Rabbit	1:300	Abcam, ab195045
Oct4	Rabbit	1:200	Cell Signaling Technology, #2750
Nanog	Rabbit	1:200	Cell Signaling Technology, #4903
SSEA-4	Mouse	1:100	Abcam, ab16287
TRA-1-60	Mouse	1:100	Abcam, ab16288

Table S2. List of used primers and probes.

Primers	Target	Forward/Reverse primer (5′-3′)
Differentiation markers (RT-PCR)	*AFP*	AAATGCGTTTCTCGTTGCTT/GCCACAGGCCAATAGTTTGT
Differentiation markers (RT-PCR)	*MSX1*	CGAGAGGACCCCGTGGATGCAGAG/GGCGGCCATCTTCAGCTTCTCCAG
Differentiation markers (RT-PCR)	*PAX6*	GTCCATCTTTGCTTGGGAAA/TAGCCAGGTTGCGAAGAACT
Differentiation markers (RT-PCR)	*FKL1*	TGATCGGAAATGACACTGGA/CACGACTCCATGTTGGTCAC
Differentiation markers (RT-PCR)	*MAP2*	CAGGTGGCGGACGTGTGAAAATTGAGAGTG/CACGCTGGATCTGCCTGGGGACTGTG
House-keeping gene (RT-PCR)	*GAPDH*	GTGGACCTGACCTGCCGTCT/GGAGGAGTGGGTGTCGCTGT
Mutated gene (RT-qPCR)	*VPS13B*	TGGAACCATCAAACAAGGCTGCA/TCCTGAGCACCACTGTAGCGA<br>FAM-BHQ1 probe - ATATTTGATGGAGGCATGGC
Reference gene (RT-qPCR)	*CAPN10*	TCAGAACCGAGTGAGGTGTA/GTATGACTGTCACCCACCAG<br>FAM-BHQ3 probe - CACGCGGCGGACTGGGCAGG
Targeted mutation analysis/Sanger sequencing (Patient 1)	*VPS13B*	chr8:g.99096425dup:<br>GGATGACCATGAAAGCTGTG/CAAAGCACTGGGATTACAGG<br>chr8:g.99481803G>T:<br>AGCAGTATAGGCACAGCTCCTC/AGGTAGGGAAAGGGGAAGC
Targeted mutation analysis/Sanger sequencing (Patient 2)	*VPS13B*	chr8:g.99832435_99832436del:<br>TGCTGTATTACTGTTGCTAATGTGC/CCTGCGACCATTGTATCTCT<br>chr8:g.99501805T>C:<br>Nested PCR primers:<br>outer:<br>GAGATTCTATACAAGCAGGTGAGG/CAGTAAAGAAGAAAAGCAATCCA<br>inner:<br>AGATTCTATACAAGCAGGTGAGG/AAAAGGAATCCTGTACCTGTGC
Targeted mutation analysis/Sanger sequencing (iPSCs)	*VPS13B*	Chr8(GRCh38.p14):g.99481803G>T<br>CAGGGCCTGTTCCTACTTCT/AGCTCCTCTTGAACCTTCAGAATC<br>Chr8(GRCh38.p14):g.99096425_99096426dup:<br>AGGATGACCATGAAAGCTGTGGT/GCTTCACCATGTTGGCCAGGCT<br>chr8:g.99832435_99832436del:<br>TTTCGTGTTCCAGACAGTGC/GGGGTACTGCCACACTCTGT<br>chr8:g.99501805T>C:<br>GCAGGTGAGGAATCACCATT/GGAGGGAGGGAAGAAAGAAA

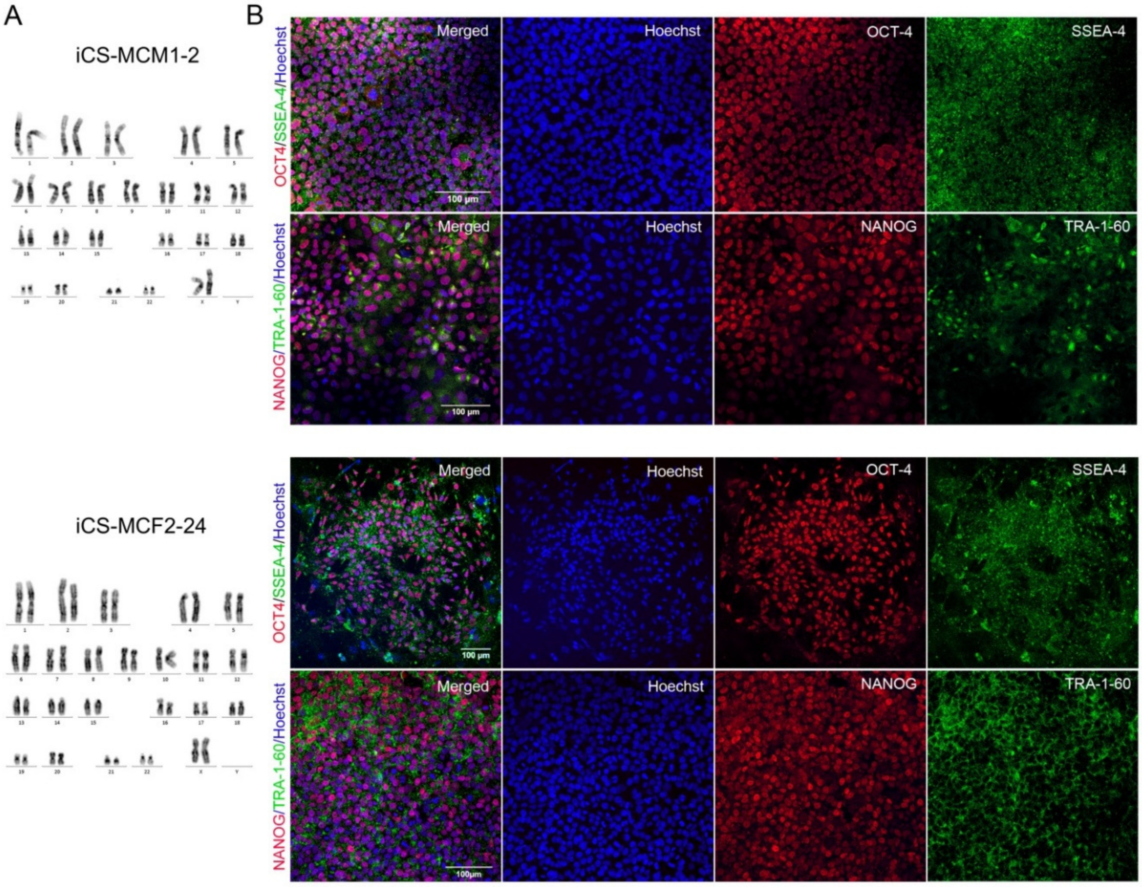

Figure S1. Characteristics of iPSC lines derived from CS patients. (A) normal karyotype (46,XX) of iPSC lines of Patient 1 - iCS-MCM1-2 and Patient 2 - iCS-MCF2-24; (B) Immunocytochemical analysis of iPSCs lines - Patient 1 - iCS-MCM1-2 and Patient 2 - iCS-MCF2-24 on the pluripotency markers with antibodies against OCT4 (red) and SSEA-4 (green), NANOG (red) and TRA-1-60 (green). The cell nuclei are counterstained with Hoechst 33258 (blue). Scale bar 100 um.

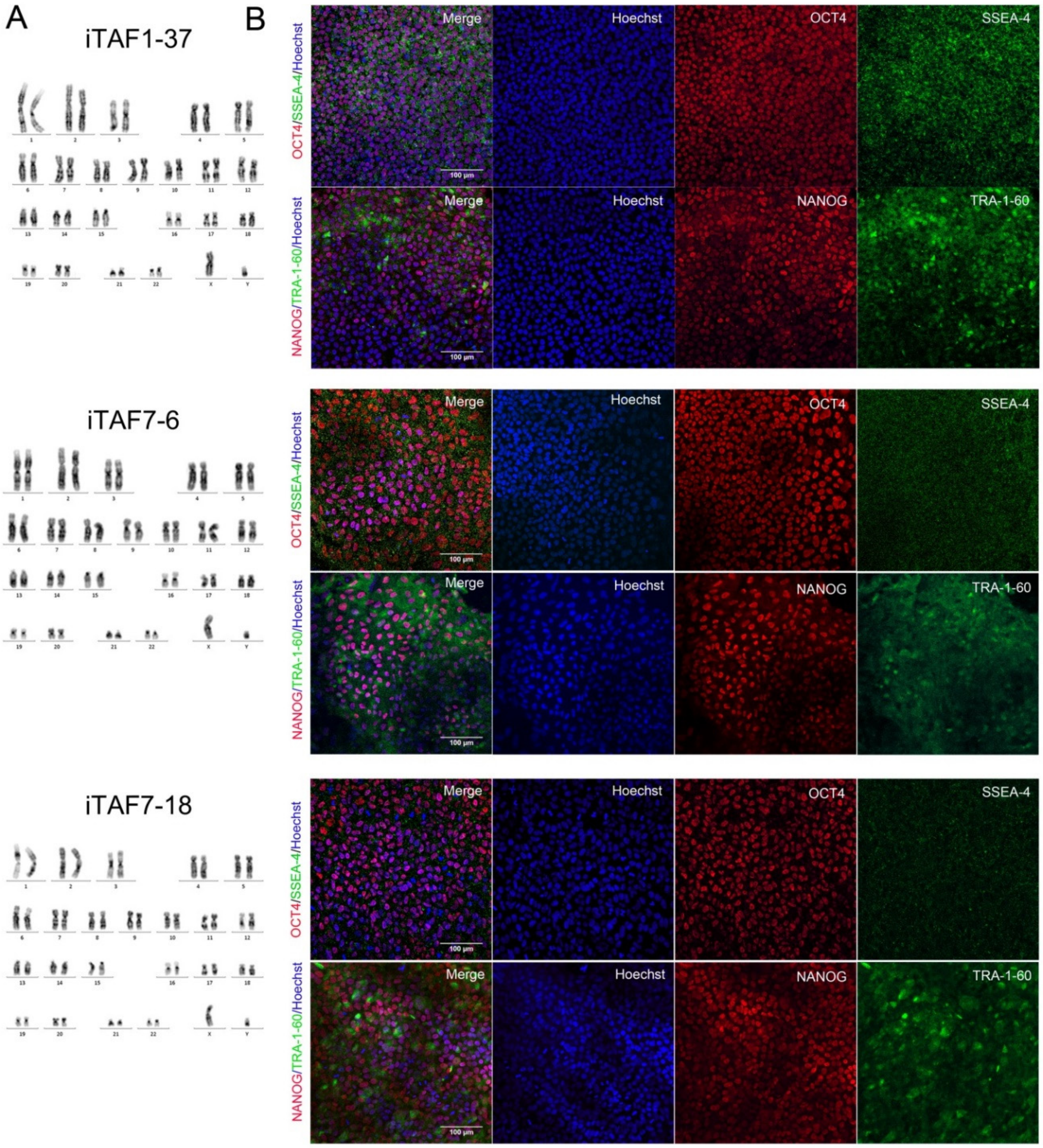

Figure S2. Characteristics of iPSC lines derived from healthy donors. (A) normal karyotype (46,XY) of iPSC lines of healthy donors - iTAF1-37, iTAF7-6, iTAF7-18. (B) Immunocytochemical analysis of iPSCs lines - iTAF1-37, iTAF7-6, iTAF7-18 on the pluripotency markers with antibodies against OCT4 (red) and SSEA-4 (green), NANOG (red) and TRA-1-60 (green). The cell nuclei are counterstained with Hoechst 33258 (blue). Scale bar 100 um.

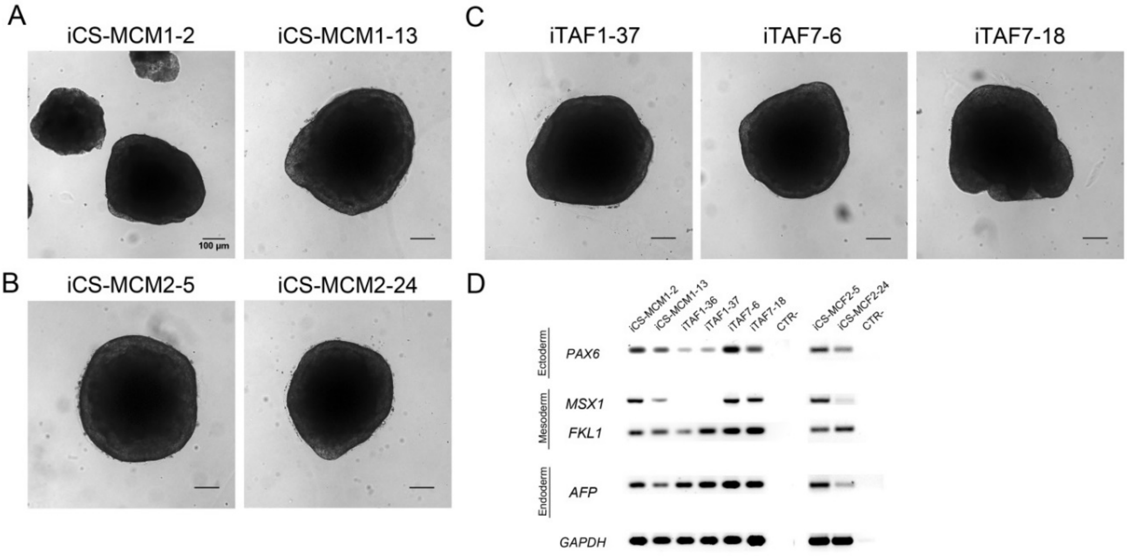

Figure S3. Characteristics of iPSC lines derived from healthy donors and CS patients for the capacity to differentiate into three germ layers. Morphology of the embryonic bodies differentiated from the (A) Patient 1 iPSC lines iCS-MCM1-2, iPSC-MCM1-13; (B) Patient 2 iPSC lines iCS-MCF2-5, iCS-MCF2-24 and (C) iPSC lines of Normal donors: iTAF1-37, iTAF7-6, iTAF7-18. (D) RT-PCR analysis of spontaneously differentiated embryonic bodies from all investigated iPSC lines revealed that markers of all three germ layers - endoderm, mesoderm, and ectoderm - were expressed in all iPSC lines generated from CS patients and normal donors.

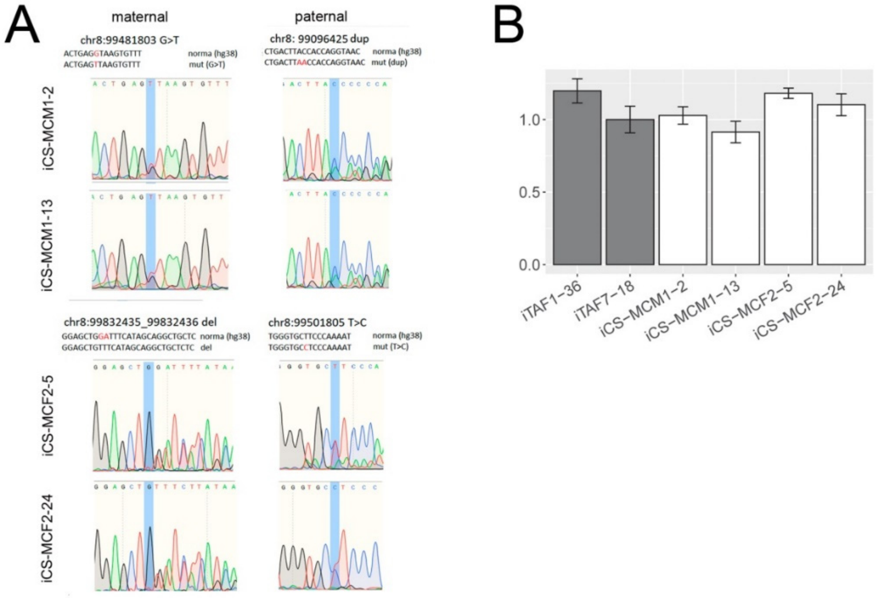

Figure S4. Analysis of iPSCs for the presents of the parental mutations and *VPS13B* expression. (A) Sanger sequencing *VPS13B* confirmed the presence of both parental mutations in iPSC lines generated from each CS patient. (B) *VPS13B* gene expression levels normalized alongside *CAPN10* mRNA. The relative level of *VPS13B* gene expression quantified by qPCR did not differ in iPSC line's proband 1 (iCS-MCM1-1 and iCS-MCM1-13), proband 2 (iCS-MCF2-5 and iCS-MCF2-24), and normal donor iPSC lines (iTAF7-18 and iTAF1-36). The data are presented as the mean ± SD.

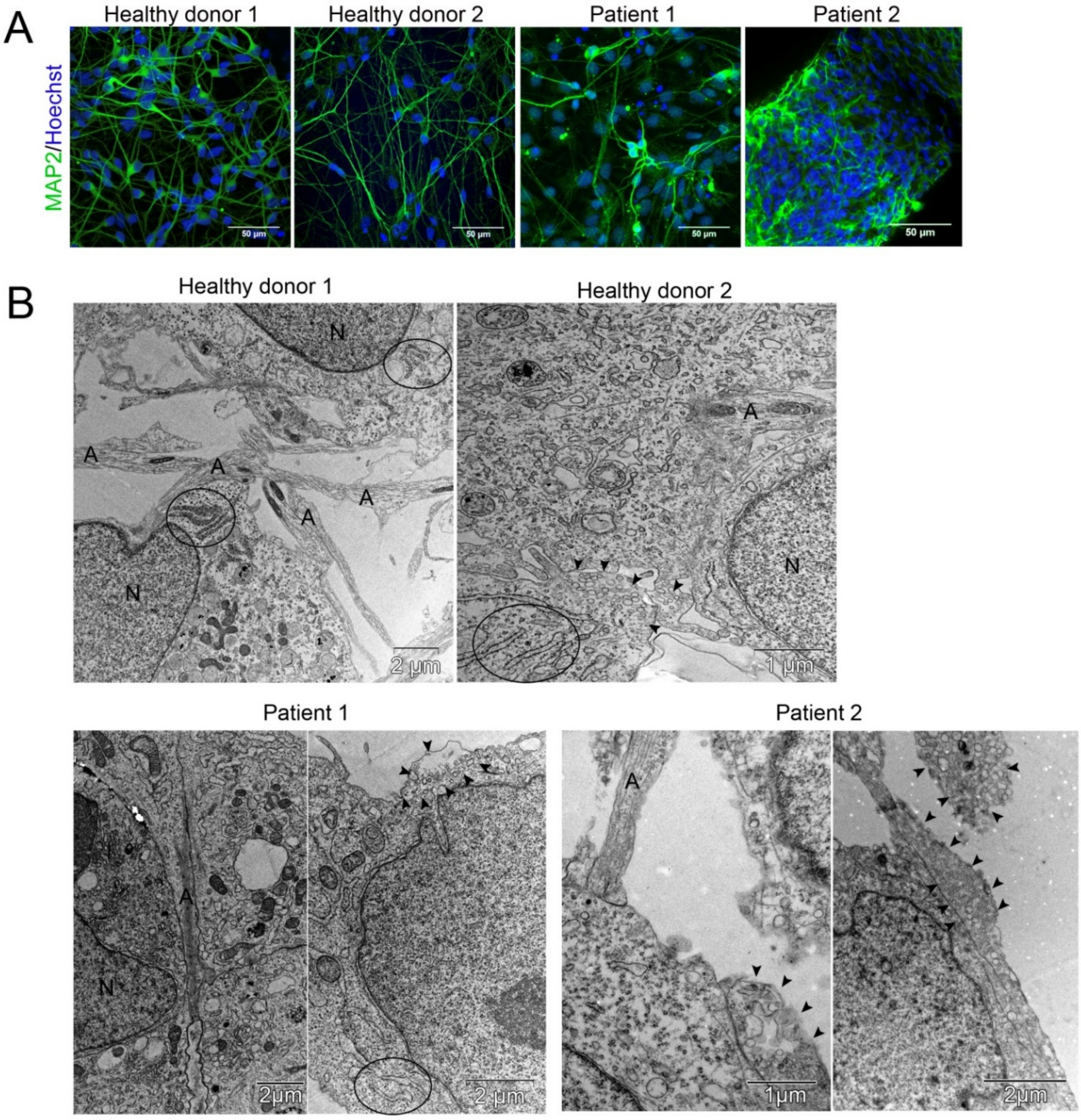

Figure S5. Characterization of neurons derived from the Healthy donors and CS-patients iPSCs. Immunocytochemical analysis of differentiated cell culture for the neuronal marker MAP2 (green) indicates successful neural network formation from the NSCs of healthy donors and CS patients. The cell nuclei are counterstained with Hoechst 33258 (blue). TEM images of iPSC-derived neurons. Both Healthy donor and CS-derived neurons possess specific features of differentiated neurons: synapses and have typical intracellular structures - neurofilaments and Nissl bodies in the cytoplasm. A - Axons, N - nucleus, Axonal terminations are marked by black arrowheads; Nissl bodies are circled.

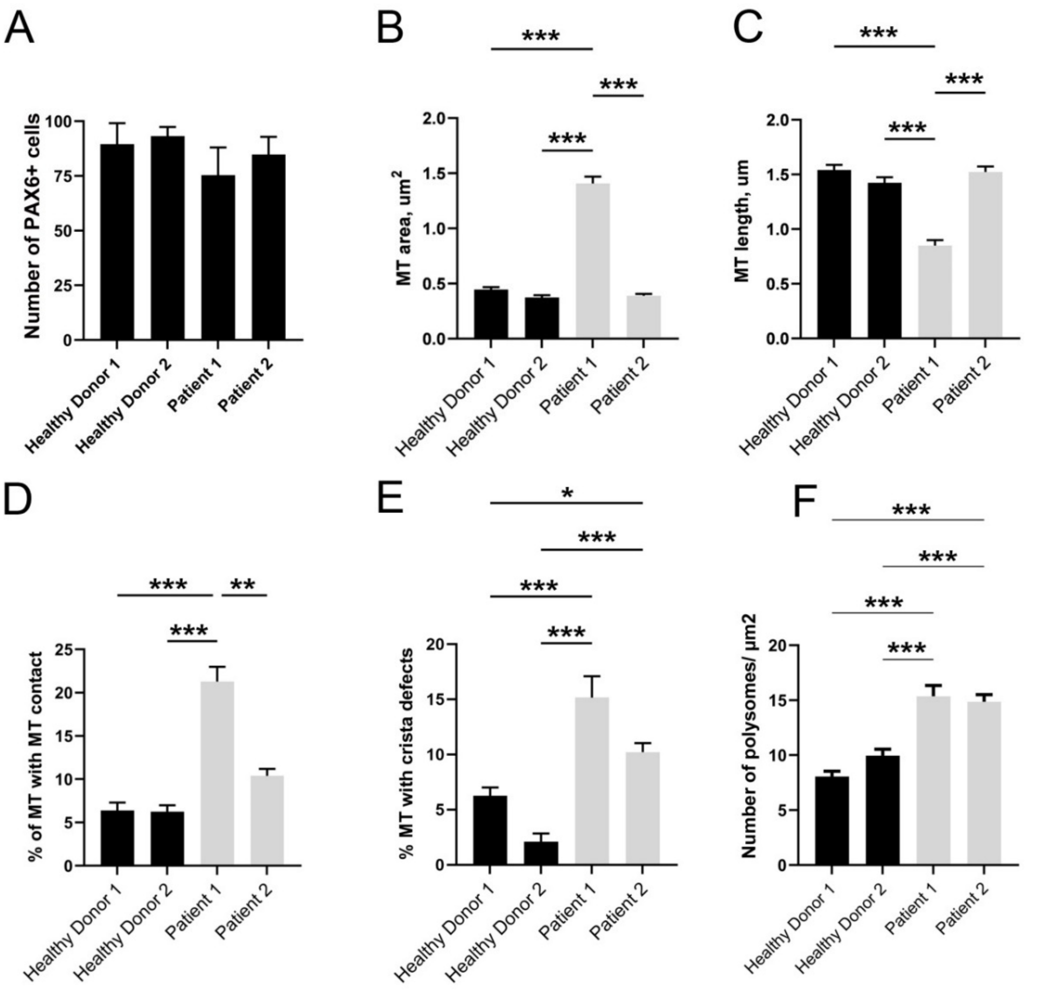

Figure S6. Morphometric analysis of differentiated NSC and neurons. Assessing the NSC differentiation efficiency of iPSCs. Quantitative analysis of PAX6+ cells in the culture of differentiated NSC. (B-E) Morphometric analysis of mitochondria in the Healthy donors and CS-patients derived neurons: morphological parameters of neuron mitochondria (MT): area (B) and length (C), (D-E). The graphs show the percentage composition per cell of MT with tight contact to another MT (D) and MT with cristae defects (E). (F) Quantitative analysis of polysomes in neurons derived from healthy donors and CS iPSC. Number of polysomes was evaluated on a randomly selected area of 1 μm2. 10 cells were randomly chosen for each donor and patient, 2 areas were evaluated in each cell. A Kruskal-Wallis test, followed by a multiple comparison test with Dunn's correction, was performed to determine statistical significance between groups (p-value ≤ 0.05). Bar plots represent the mean ± SEM. Statistically significant differences between groups are displayed as: * = p-value < 0.05, ** = p-value < 0.01, *** = p-value < 0.001.
